# Toward Precision Imaging in Lung NET: A Clinically Oriented Framework Integrating Dual Tracer PET and Radiomics

**DOI:** 10.3390/cancers18193111

**Published:** 2026-09-25

**Authors:** Anna La Salvia, Roberta Elisa Rossi, Piero Paravani, Enrico D’Ippolito, Arianna Gagliardi, Giorgia Maria Granese, Rosaria Meucci, Luciano Carideo, Davide Campana, Alberto Signore, Annamaria Colao, Antongiulio Faggiano, Daniela Prosperi, NIKE Group

**Affiliations:** 1 National Center for Drug Research and Evaluation, National Institute of Health (ISS), 00161 Rome, Italy; 2 Department of Biomedical Sciences, Humanitas University, Via Rita Levi Montalcini 4, Pieve Emanuele, 20072 Milan, Italy; roberta.elisa.rossi@hunimed.eu; 3 Gastroenterology and Endoscopy Unit, IRCCS Humanitas Research Hospital, Rozzano, 20089 Milan, Italy; 4 Endocrinology Unit, Department of Clinical and Molecular Medicine, Sant’Andrea Hospital, ENETS Center of Excellence, Sapienza University of Rome, 00189 Rome, Italy; piero.paravani@uniroma1.it (P.P.); antongiulio.faggiano@uniroma1.it (A.F.); 5 Nuclear Medicine Unit, Department of Medical-Surgical Sciences and of Translational Medicine, Faculty of Medicine and Psychology, “Sapienza” University, 00189 Rome, Italy; enrico.dippolito@uniroma1.it (E.D.); granese.2092337@studenti.uniroma1.it (G.M.G.); rosaria.meucci@ptvonline.it (R.M.); alberto.signore@uniroma1.it (A.S.); 6 Department of Experimental Medicine, Sapienza University of Rome, 00185 Rome, Italy; arianna.gagliardi@uniroma1.it; 7 U.O.C. Diagnostic Imaging, PTV Policlinico “Tor Vergata” University, 00133 Rome, Italy; 8 Nuclear Medicine Unit, University Hospital Sant’Andrea, 00189 Rome, Italy; lcarideo@ospedalesantandrea.it (L.C.); dprosperi@ospedalesantandrea.it (D.P.); 9 Medical Oncology Unit, Department of Medical and Surgical Sciences (DIMEC), Alma Mater Studiorum—University of Bologna, 40138 Bologna, Italy; davide.campana@unibo.it; 10 Department of Clinical Medicine and Surgery, Section of Endocrinology, Diabetology, Andrology and Nutrition, University of Naples Federico II, 80138 Naples, Italy; annamaria.colao@unina.it; 11 UNESCO Chair for Health Education and Sustainable Development, University of Naples Federico II, 80138 Naples, Italy

**Keywords:** lung NET, NEN, nuclear medicine, radiopharmaceuticals, PET/CT, radiological imaging, radiomics, precision imaging, FDG, SSTR

## Abstract

**Background**: Pulmonary neuroendocrine tumors (lung NETs) comprise a biologically heterogeneous group of neoplasms ranging from indolent typical carcinoids to more aggressive atypical carcinoids. Accurate characterization of tumor biology is essential for optimizing staging, prognostic stratification, and treatment selection. Beyond conventional cross-sectional imaging, functional imaging and emerging quantitative imaging techniques are progressively redefining the diagnostic pathway. **Methods**: We performed a comprehensive narrative review of the current evidence regarding conventional radiological imaging, somatostatin receptor (SSTR) PET/CT, ^18^F-FDG PET/CT, dual-tracer imaging, and radiomics in lung NETs, integrating recent international recommendations from ENETS, ESMO, AIOM, and other major societies. **Results**: Contrast-enhanced CT remains the cornerstone of anatomical staging, whereas ^68^Ga-labelled somatostatin analogue (SSA) PET/CT provides highly sensitive assessment of receptor expression and patient eligibility for somatostatin analogue therapy and peptide receptor radionuclide therapy (PRRT). Conversely, ^18^F-FDG PET/CT identifies metabolically active and biologically aggressive disease, particularly in atypical carcinoids and tumors showing dedifferentiation. Increasing evidence supports the complementary role of dual-tracer PET/CT for non-invasive characterization of tumor heterogeneity, prognostic stratification, and treatment planning. Radiomics further expands this paradigm by extracting quantitative imaging biomarkers that may improve histological prediction, recurrence risk assessment, and individualized management. Although promising, radiomics remains limited by methodological heterogeneity and the lack of prospective validation. **Conclusions**: The integration of conventional imaging, molecular imaging, and quantitative radiomics supports a shift from lesion detection toward biologically driven precision imaging. Future prospective multicenter studies incorporating imaging biomarkers, artificial intelligence, and clinical-pathological variables may further support personalized management of patients with lung NETs.

## Simple Summary

Lung neuroendocrine tumors are rare cancers with highly variable biological behavior, ranging from slow-growing tumors to more aggressive forms. Accurate characterization is essential to guide treatment and improve patient outcomes. Conventional radiological imaging, contrast-enhanced CT, remains the cornerstone for anatomical staging, but it provides limited information on tumor biology. Molecular imaging with somatostatin receptor PET/CT and ^18^F-FDG PET/CT offers complementary insights into receptor expression, metabolic activity, and tumor aggressiveness, supporting prognosis and treatment selection, including eligibility for peptide receptor radionuclide therapy. Emerging quantitative techniques such as radiomics further enhance image analysis by extracting imaging biomarkers associated with tumor heterogeneity and clinical outcomes. This review summarizes current evidence and international recommendations on multimodal imaging in lung NETs, highlighting how the integration of anatomical, molecular, and quantitative imaging may improve disease characterization and support a more personalized, precision medicine approach for patient management.

## 1. Introduction

Pulmonary neuroendocrine neoplasms (NENs) are rare malignant tumors characterized by neuroendocrine morphology and expression of markers such as chromogranin A and synaptophysin. According to the 2021 WHO classification, well-differentiated pulmonary neuroendocrine tumors (NETs) include typical carcinoid (TC; <2 mitoses/2 mm^2^ and absence of necrosis), atypical carcinoid (AC; 2–10 mitoses/2 mm^2^ and/or focal necrosis), and carcinoid/NET with elevated mitotic counts and/or Ki-67 proliferation index (>10 mitoses/2 mm^2^ or >30% Ki-67), which displays intermediate features between AC and neuroendocrine carcinoma and more aggressive behavior [1,2]. Although Ki-67 is widely used as a prognostic and complementary diagnostic marker, its role in pulmonary NET grading remains controversial, and it is not currently incorporated into the WHO grading system. Of note, pulmonary NENs comprise a spectrum of tumors, including well-differentiated NETs and poorly differentiated neuroendocrine carcinomas, including large-cell neuroendocrine carcinoma (LCNEC) and small-cell lung cancer (SCLC). The present review focuses primarily on well-differentiated pulmonary NETs, particularly typical and atypical carcinoids. The broader term pulmonary NENs is used when referring to studies or concepts that include multiple neuroendocrine histological subtypes.

Lung NETs account for approximately 1–2% of lung malignancies and about 30% of all NETs. Their incidence and prevalence have increased over recent decades, likely reflecting improved diagnostic capabilities and greater clinical awareness [1,3,4]. A slight female predominance has been reported, with no established risk factors [5]. Histological subtype and pathological stage, particularly lymph node involvement, are the main validated prognostic factors. Tumor size, mitotic count, necrosis, Ki-67 index, completeness of surgical resection, tumor growth and overall tumor burden also contribute to risk stratification [1,2]. More recently, multivariable prognostic models integrating these clinicopathological features, including nomograms combining histological subtype and TNM stage, have improved prediction of overall and progression-free survival, highlighting the biological heterogeneity of lung NETs beyond the traditional TC/AC classification [6].

Initial staging is essential to guide diagnosis, treatment and precision medicine. Conventional radiology, functional imaging and emerging techniques such as radiomics provide complementary information in this heterogeneous disease. Staging generally follows the TNM classification for lung cancer, although its prognostic applicability to carcinoid tumors has been less extensively validated than in conventional lung carcinomas, and no specific staging system currently exists for these tumors [1,7].

Contrast-enhanced computed tomography (CT) remains the cornerstone of initial staging, enabling assessment of the primary tumor, bronchovascular involvement, lymph nodes, and distant metastases. Pulmonary NETs express somatostatin receptors (SSTRs), predominantly SSTR2, which are generally higher and more homogeneous in TC and reduced or heterogeneous in AC and highly proliferative NETs. This receptor expression supports the use of radiolabelled somatostatin analogues for functional imaging. ^68^Ga-DOTA-peptide PET/CT enables sensitive detection of SSTR-positive disease, whole-body staging and assessment of receptor status, supporting selection for somatostatin analogues (SSAs) and peptide receptor radionuclide therapy (PRRT). Conversely, ^18^F-FDG PET/CT reflects proliferative activity and aggressive tumor behavior and is particularly useful in AC, highly proliferative NETs, discordant imaging findings and rapidly progressive disease, providing complementary prognostic information [7,8,9].

Surgical resection with systematic lymph node dissection is the treatment of choice for resectable local and locoregional disease and is associated with favorable long-term outcomes, particularly in TC. Advanced or unresectable disease requires multidisciplinary management, including SSAs, targeted therapies such as everolimus, chemotherapy and PRRT with ^177^Lu-DOTATATE in selected patients [1,10,11]. Given the increasing complexity of diagnostic pathways and the expanding role of molecular imaging, this review examines conventional and functional imaging techniques, including radiomics, and their complementary role in an integrated clinical framework for optimizing the diagnosis, staging and management of lung NETs.

## 2. Materials and Methods

We conducted a comprehensive narrative review of the available evidence on conventional radiological imaging, SSTR PET/CT, ^18^F-FDG PET/CT, dual-tracer imaging, and radiomics in pulmonary neuroendocrine tumors. The literature search was conducted in PubMed/MEDLINE, Scopus and Web of Science for publications available up to July 2026. The search strategy combined terms related to pulmonary neuroendocrine tumors, lung carcinoids, SSTR PET/CT, 18F-FDG PET/CT, dual-tracer imaging, radiomics, prognosis, staging, and treatment selection. Relevant original studies, systematic reviews, consensus documents, clinical practice guidelines, and selected methodological papers were considered. Studies were included when they addressed the diagnostic, staging, prognostic, or treatment-related implications of imaging techniques in pulmonary neuroendocrine tumors. Publications that were unrelated to pulmonary neuroendocrine neoplasms, did not provide relevant information on the imaging modalities under consideration, or were not available in an appropriate publication format were excluded. The review integrated recent international recommendations and guidelines from major scientific societies, including the European Neuroendocrine Tumor Society, the European Society for Medical Oncology, and the Italian Association of Medical Oncology.

Because of the substantial heterogeneity in study populations, histological subtypes, imaging protocols, tracer administration, quantitative parameters, outcome measures, and study designs, the available evidence was synthesized narratively rather than through a formal quantitative meta-analysis. The review was intended to provide a clinically oriented overview of the complementary role of anatomical imaging, molecular imaging, and radiomics, while identifying areas requiring prospective validation.

## 3. Results

The results are organized according to the principal clinical applications of imaging in pulmonary NETs: diagnosis and characterization, staging and risk assessment, and treatment selection and follow-up. The available evidence regarding individual imaging modalities is first discussed, followed by an integrated assessment of their potential clinical applications and current recommendations. Particular attention is given to the complementary information provided by anatomical imaging, SSTR PET/CT, 18F-FDG PET/CT, dual-tracer imaging, and radiomics.

### 3.1. Nuclear Medicine Radiopharmaceuticals

#### 3.1.1. ^68^Ga-SSA PET/CT

The clinical introduction of 111In-pentetreotide scintigraphy, followed by 99mTc-HYNIC-TOC, represented the first successful applications of SSTR-targeted imaging in NENs. However, their diagnostic performance was limited by the suboptimal spatial resolution and sensitivity of single-photon emission computed tomography (SPECT). The advent of ^68^Ge/^68^Ga generator technology enabled the development of PET/CT with ^68^Ga-labelled SSAs, markedly improving image quality, lesion detectability, acquisition efficiency, and diagnostic accuracy and establishing SSTR PET/CT as the reference standard for functional imaging of well-differentiated NENs [12,13,14,15,16,17,18,19].

The most commonly used tracers, ^68^Ga-DOTA-TATE, ^68^Ga-DOTA-TOC, and ^68^Ga-DOTA-NOC, show comparable diagnostic performance despite minor differences in receptor affinity. Among these tracers, 68Ga-DOTATATE has particular theranostic relevance because the same somatostatin receptor-targeting principle can be exploited for diagnostic imaging and peptide receptor radionuclide therapy using ^177^Lu-DOTATATE. More broadly, the theranostic concept does not require identical molecular structures, but rather relies on the use of compatible agents targeting a shared biological pathway or receptor system for patient selection and treatment [20,21]. International guidelines recommend ^68^Ga-DOTA-peptide PET/CT for staging, restaging, characterization of indeterminate bronchial lesions, and identification of occult primary neuroendocrine tumors [12,22].

Beyond lesion detection, ^68^Ga-SSA PET/CT provides biological information because tracer uptake reflects tumor differentiation. The relationship between histological subtype and SSTR expression is clinically relevant but not absolute. TCs generally demonstrate more intense and homogeneous SSTR expression, whereas ACs may show lower or more heterogeneous uptake, reflecting greater biological variability. However, substantial inter-tumoral and intra-tumoral heterogeneity may occur within each histological category. Accordingly, SSTR PET/CT should not be interpreted as a surrogate for histological classification alone, but rather as a complementary tool for assessing receptor expression, disease distribution, and potential eligibility for receptor-targeted therapies. In patients with discordant imaging findings, aggressive clinical behavior, or suspected dedifferentiation, complementary 18F-FDG PET/CT may provide additional information regarding metabolic activity and tumor heterogeneity [12]. Quantitative parameters such as SUVmax may also reflect tumor phenotype, with higher values reported in TCs than in ACs [23]. Accordingly, ^68^Ga-SSA PET/CT has become the reference functional imaging modality for pulmonary carcinoids (PCs), and the 2021 European Society for Medical Oncology (ESMO) guidelines recommend its integration with contrast-enhanced CT for TNM staging and treatment planning [1].

Early clinical evidence was provided by Jindal et al. [24], who reported a 95% detection rate for ^68^Ga-DOTA-TOC PET/CT in 20 patients with primary lung NETs. PET/CT identified additional disease not detected by conventional imaging in one patient, supporting its value for staging. Walker et al. [25] subsequently demonstrated markedly higher ^68^Ga-DOTA-TATE uptake in carcinoids than in non-neuroendocrine lung cancers, reflecting high SSTR2 expression in well-differentiated NETs. Although inflammatory lesions could also show tracer uptake, limiting specificity for benign versus malignant pulmonary nodules, the findings supported a complementary role for SSTR and ^18^F-FDG PET/CT in lesion characterization.

Lamarca et al. [26] evaluated ^68^Ga-DOTA-NOC PET/CT in 46 patients with lung NETs across different clinical settings, including diagnosis, staging, postoperative assessment, primary tumor localization, and PRRT eligibility. PET findings influenced management in approximately one-third of patients by modifying staging, guiding treatment, or identifying PRRT candidates, with the greatest benefit observed in metastatic or recurrent disease. More recently, Rufini et al. [27] demonstrated a significant correlation between ^68^Ga-DOTA-peptide uptake and immunohistochemical SSTR2 expression, with higher uptake in typical than atypical carcinoids, further supporting the role of SSTR PET/CT in assessing tumor differentiation and selecting patients for PRRT.

Overall, these studies established ^68^Ga-DOTA-peptide PET/CT as a valuable modality for lung NETs, providing accurate staging and information on SSTR expression with direct implications for treatment selection and PRRT eligibility. The 2025 ENETS Guidelines [28] further recognize ^68^Ga-labelled SSA PET/CT as the preferred functional imaging modality for well-differentiated pulmonary NETs, including TC and AC. It is particularly recommended at diagnosis and in potentially resectable disease, in combination with contrast-enhanced CT or MRI, to improve the detection of regional lymph-node and distant metastases. Importantly, high tracer uptake generally indicates well-differentiated disease and identifies patients more likely to benefit from SSAs and PRRT; adequate SSTR expression on ^68^Ga-SSA PET/CT is therefore an essential prerequisite before PRRT.

From a clinical perspective, the assessment of SSTR expression may contribute to treatment planning, particularly when receptor-targeted approaches are being considered. However, the presence and intensity of SSTR uptake should be interpreted together with histological findings, disease extent, tumor growth kinetics, prior treatments, and the presence of lesions with discordant metabolic or receptor-based imaging characteristics. In this context, SSTR PET/CT provides functional information that complements rather than replaces pathological assessment and conventional anatomical imaging.

#### 3.1.2. ^18^F-FDG PET/TC

^18^F-FDG, the most widely used radiopharmaceutical in oncological PET imaging, is a glucose analogue taken up via GLUTs, which are frequently overexpressed in malignant tissues. Following phosphorylation by hexokinase, ^18^F-FDG -6-phosphate is retained intracellularly, enabling non-invasive assessment of tumor glycolytic activity [29]. A strong correlation between GLUT-1 expression and ^18^F-FDG uptake has been reported in lung cancer [30]. In NENs, its role remains debated in well-differentiated tumors [31,32], whereas high-grade tumors generally show increased GLUT expression and glucose metabolism, often with reduced SSTR expression; consequently, higher ^18^F-FDG uptake is associated with aggressive disease and poorer prognosis [33,34]. The administered activity of 18F-FDG varies according to institutional protocols, patient characteristics, scanner specifications, and acquisition procedures. In the studies considered in this review, imaging was generally performed approximately 60 min after tracer administration in fasting, euglycemic patients. The activity reported in individual studies should therefore be interpreted in the context of the original study protocol and should not be considered a universally recommended dose [12,35].

^18^F-FDG is not tumor-specific, as inflammatory and infectious processes may also cause increased uptake through leukocyte glycolysis. Marked accumulation has been described in lung collapse due to obstructive pneumonia secondary to endobronchial carcinoids [36,37], whereas ^68^Ga-SSA showed minimal uptake in the same areas [38,39].

^18^F-FDG uptake can be quantitatively assessed using SUVmax and SUVmean, while MTV and TLG may provide additional information in selected settings.

^18^F-FDG PET/CT is also routinely incorporated into the diagnostic and staging work-up of many patients with suspected or confirmed lung cancer, depending on the clinical setting, disease extent, and pretest probability. Consequently, FDG PET/CT may already be available as part of the broader oncological evaluation of patients with pulmonary neuroendocrine tumors. In this context, its complementary value in lung NETs should be interpreted in relation to histological subtype, SSTR expression, clinical behavior, and the information provided by conventional and SSTR-based imaging.

In lung NETs, Jiang et al. [23] identified an SUVmax cutoff of 3.7 for distinguishing TCs from ACs (sensitivity 73.9%, specificity 65.4%), although the modest AUC (73.3%) limits its diagnostic value. Similarly, other studies found SUVmax unreliable for differentiating ACs from TCs [12,27]. However, the SUVmax/SUVliver ratio correlates with Ki-67 in lung NETs and may identify ACs with higher proliferation (Ki-67 10–20%) [40,41]. Briganti et al. suggested ^18^F-FDG PET/CT during follow-up, as new or increasing uptake may indicate dedifferentiation and disease progression [40].

Bozkurt et al. recommend ^18^F-FDG for localization and management of high-grade, poorly differentiated NENs and as a prognostic marker [22]. The 2021 ESMO guidelines recommend it in ACs with high-grade histology, particularly when ^68^Ga-SSA PET/CT is negative [1]. Ambrosini et al. recommend combining ^18^F-FDG with ^68^Ga-SSA to assess tumor aggressiveness, particularly at baseline, in rapid progression despite low-grade histology, for CT/MRI-positive but ^68^Ga-SSA-negative lesions, prognostic assessment, and identification of mismatched ^18^F-FDG-positive/68Ga-SSA-negative lesions [35].

According to the 2025 ENETS guidance, Baudin et al. suggest a complementary role for ^18^F-FDG PET/CT in selected patients with low or absent SSTR expression. Although its prognostic value in lung NETs remains unconfirmed, it may be considered for surveillance when it provides incremental diagnostic information over ^68^Ga-SSA PET/CT at initial staging.

#### 3.1.3. Combined Imaging with ^68^Ga-SSA and ^18^F-FDG PET/CT

Functional imaging has increasingly improved the evaluation of pulmonary carcinoids through the complementary use of ^68^Ga-labelled SSA and ^18^F-FDG PET/CT, allowing simultaneous assessment of SSTR expression and tumor glycolytic activity [42]. This dual-tracer approach is particularly relevant given the biological heterogeneity of lung NETs.

Early studies demonstrated distinct imaging profiles, with higher SSA uptake in typical carcinoids (TCs) and higher FDG uptake in atypical carcinoids (ACs) [43,44]. Prospective and multicenter studies confirmed superior sensitivity of SSA PET/CT for TCs and of FDG PET/CT for ACs, with detection rates ranging from 91 to 100% versus 35–50% depending on histology [45,46]. SUV ratios (SUVr) also appeared more informative than SUVmax alone, with a reported cutoff of 1.19 providing >80% sensitivity and 90% specificity for distinguishing TC from AC. An inverse correlation between FDG and SSA uptake was also described [47]. Overall, these findings support the paradigm of TC = SSA-high/FDG-low and AC = FDG-high/SSA-low, suggesting that dual-tracer imaging may improve preoperative tumor phenotyping and risk assessment.

Several studies have proposed SUV-based thresholds or SUV ratios to differentiate typical from atypical carcinoids or to characterize tumor biology. However, these values were derived from individual cohorts and specific imaging protocols. Their diagnostic performance may be influenced by differences in patient selection, tracer administration, acquisition parameters, image reconstruction, lesion characteristics, and analytical methodology. Accordingly, the reported thresholds should be interpreted as study-specific findings rather than as universally validated clinical decision limits. Their potential clinical application requires confirmation in larger, prospective and methodologically standardized cohorts.

Lococo et al. [48] further evaluated both tracers in 62 patients (55 TCs, 7 ACs). Overall, SSA PET/CT showed higher detection rates than FDG (88.4% vs. 53.8% using an SUVmax cutoff of 2.5), increasing to 100% versus 80.8% with a cutoff of 1.5. However, this advantage was mainly observed in TCs, whereas FDG provided greater accuracy in ACs. Positive SSA uptake was associated with low mitotic activity, while negative scans were more frequently observed in tumors with necrosis.

Dual-tracer imaging may also have therapeutic implications. Zidan et al. [49], in 56 well-differentiated PCs, identified four molecular profiles based on ^68^Ga-DOTA-TATE and FDG uptake. Half of the patients showed SSA-positive profiles and were considered potential candidates for PRRT. Intratumoral heterogeneity occurred in 34% of cases. Among 16 patients treated with PRRT, excluding one palliative case, disease control was achieved in 85%, including 46% partial responses and 39% stable disease [49]. These findings suggest that dual-tracer PET/CT may help identify patients most likely to benefit from radionuclide therapy.

Deleu et al. [50] similarly found higher SSA SUVmax in TCs and higher FDG SUVmax in ACs. SSA PET/CT also demonstrated good performance for nodal and distant metastatic assessment, with 80% sensitivity and 75% specificity for hilar-mediastinal nodes at an SUVmax cutoff of 2.1, while all distant lesions were detected and biopsy-confirmed, yielding a PPV of 100%.

In a multicenter study of 61 patients, Albano et al. [51] confirmed the complementary biological information provided by the two tracers. An SSA-to-FDG SUVr cutoff of 1.05 differentiated TC from AC with an AUC of 0.889. Histology remained the only independent predictor of PFS and OS, although FDG and SSA positivity were independently associated with PFS, while SUVr correlated with OS on univariate analysis. Thus, SUVr may be particularly useful preoperatively when histology is unavailable.

Nogareda Seoane et al. [52] also supported the value of combined ^68^Ga-DOTA-TOC and FDG PET/CT in neuroendocrine tumors, with SSTR-dominant uptake associated with well-differentiated disease and FDG avidity reflecting higher-grade biology, although pulmonary NETs were not analyzed separately.

The 2025 ENETS guidelines [28] emphasize that ^68^Ga-SSTR and ^18^F-FDG PET/CT provide complementary rather than alternative information on tumor differentiation and aggressiveness. Dual-tracer imaging may be considered in selected patients with ACs, rapidly progressive or discordant disease, or suspected dedifferentiation. Follow-up should be individualized, particularly when recurrence, equivocal conventional imaging, progression, or eligibility for PRRT/systemic treatment is suspected.

Overall, dual-tracer PET/CT offers a more comprehensive, biologically driven characterization of lung NETs, improving preoperative differentiation between TC and AC and potentially supporting risk stratification and treatment selection. Nevertheless, its integration into routine clinical practice remains incompletely defined and requires further prospective validation.

The main findings from studies evaluating ^68^Ga-SSA PET/CT, ^18^F-FDG PET/CT, and their combined use in pulmonary NETs are summarized in Table 1. The table integrates the reported diagnostic performance, associations with histological subtype and tumor biology, and potential clinical applications, including staging, risk assessment, treatment selection, and evaluation of eligibility for PRRT. Given the heterogeneity of the study populations, imaging protocols, and quantitative parameters, reported SUV-based thresholds should be interpreted cautiously and are not considered universally applicable. Overall, the available evidence supports a complementary role for SSTR and FDG PET/CT, particularly in characterizing the biological heterogeneity of pulmonary NETs and informing individualized clinical decision-making.

### 3.2. Conventional Radiological Imaging and Radiomics

Radiomics involves the high-throughput extraction and analysis of quantitative features from medical images, based on the premise that imaging contains information beyond visual assessment [53]. The workflow generally includes five stages: data selection, image acquisition, feature extraction, exploratory analysis, and predictive modelling [54]. When integrated with clinical, pathological, and molecular data, radiomic features (RFs) may support personalized clinical decision-making. Interest in radiomics for pulmonary NENs has increased, with early work by Thuillier et al. exploring whether ^18^F-FDG PET/CT parameters and RFs could differentiate histological subtypes [55].

Paravani et al. investigated radiomic predictors of postoperative recurrence in lung NETs. Three features were significantly associated with recurrence: DependenceEntropy (GLDM, *p* = 0.049), DependenceNonUniformityNormalized (*p* = 0.024; AUC 0.796, 95% CI 0.649–0.901), and a 3D shape feature (*p* = 0.039; AUC 0.817, 95% CI 0.674–0.916) [56]. However, their RFs were extracted from unenhanced chest CT. Since non-contrast CT may provide limited additional information beyond anatomical assessment, post-contrast phases are generally considered important, particularly for tumor staging [57,58]. Consistently, Cozzi et al. identified significant associations between contrast-enhanced CT textural features, including Skewness and ClusterShade, Ki-67 classes, and metastatic disease; notably, Skewness values were negative in patients with metastases and approximately zero in those without metastases [57].

Liu et al. investigated the differential diagnosis of peripheral solid pulmonary nodules, including 201 NENs (38 NETs, 41 LCNECs, and 122 SCLCs) and adenocarcinomas, using conventional CT features and radiomics [59]. Three models were compared: a conventional CT model (ROC-AUC = 0.729), a radiomics model (ROC-AUC = 0.787), and a combined model incorporating radiological and radiomic features, which achieved the highest performance (ROC-AUC = 0.807). These findings support radiomics as a potential non-invasive tool for pulmonary tumor classification, although the inclusion of a limited range of histotypes restricts generalizability. Similarly, Borisov et al. developed a multicenter CT-based radiomic model distinguishing NENs from non-small cell lung cancer, achieving high diagnostic accuracy [60]. Other studies have explored radiomics for differentiating pulmonary NENs from hamartomas [58,61,62,63], an important distinction given their potentially overlapping CT appearances but substantially different biological behavior, prognosis, and treatment.

Overall, radiomics may provide quantitative biomarkers that complement conventional imaging and improve tumor classification, prognostic assessment, and characterization of biological aggressiveness. However, its clinical translation remains limited by methodological heterogeneity and the lack of standardized evaluation criteria. The Radiomics Quality Score (RQS) proposed by Lambin et al. provides an important framework for assessing the methodological quality and reproducibility of radiomic studies [64]. Despite advances in deep-learning-based feature extraction, most available studies remain constrained by relatively small cohorts. Larger, homogeneous, and externally validated datasets, together with standardized acquisition and reporting protocols, are therefore required to establish robust and clinically applicable radiomic models.

### 3.3. Clinical Evidence: Indications and Guidelines

#### 3.3.1. Imaging for Diagnosis and Characterization

The clinical interpretation of imaging findings in lung NETs should be guided by the specific clinical scenario, including initial diagnosis and characterization, staging, risk assessment, evaluation of recurrence or disease progression, treatment selection, and follow-up. Anatomical and functional imaging modalities, including conventional radiology, SSTR PET/CT, and 18F-FDG PET/CT, provide complementary information on disease extent and tumor biology. In selected patients, dual-tracer imaging may further contribute to the assessment of intra- and inter-lesional heterogeneity and support individualized clinical decision-making. The choice of imaging modality should be tailored to the histological subtype, disease behavior, and specific diagnostic or therapeutic objectives. This section summarizes the principal recommendations from available guidelines and consensus documents, while highlighting areas in which the evidence remains limited and further prospective validation is required.

The management of lung NETs requires an integrated evaluation of clinical, pathological, anatomical, and functional features, reflecting the marked biological heterogeneity of these neoplasms. Although the WHO classification and TNM staging system remain the cornerstone of prognostic assessment, recent evidence and international recommendations increasingly recognize additional biological parameters, including tumor growth rate, tumor burden, functional imaging characteristics, and molecular phenotypes, to better define disease behavior and support individualized therapeutic strategies [1,28,65,66]. In this context, functional imaging has progressively evolved from tumor localization and staging toward non-invasive tumor phenotyping, assessment of intra-patient heterogeneity, and characterization of biological aggressiveness, supporting the concept of precision imaging [54,67].

#### 3.3.2. Imaging for Staging and Risk Assessment

According to the most recent ENETS guidance for lung and thymic carcinoids, accurate TNM staging should rely on multiphasic contrast-enhanced computed tomography and somatostatin receptor imaging (SRI) PET, preferably performed with ^68^Ga-labelled SSAs [28]. Similarly, the ESMO guidelines identify contrast-enhanced cross-sectional imaging and SSTR PET/CT as the basis of diagnostic evaluation in lung carcinoids [1].

The central role of SSTR PET/CT reflects the frequent expression of SSTR, particularly SSTR2, in well-differentiated lung NETs, allowing assessment of disease distribution, receptor status, and eligibility for receptor-targeted treatments, including PRRT [65]. ENETS 2025 guidance further highlights the prognostic value of SSTR expression assessed by PET, as homogeneous and intense uptake is generally associated with a more favorable tumor phenotype [28]. Beyond localization, SSTR PET defines an imaging phenotype reflecting neuroendocrine differentiation and receptor density, with direct therapeutic implications because preserved receptor expression identifies patients potentially suitable for PRRT while also providing prognostic information [68]. However, SSTR expression may vary among lesions and within individual patients, and increasing evidence indicates that such heterogeneity may limit receptor-based imaging alone [28,69]. Inter-lesional heterogeneity may explain heterogeneous treatment response and cannot be adequately assessed by histological sampling alone, emphasizing the value of whole-body molecular imaging [42,69]. In selected patients, SRI-PET may underestimate disease extent, particularly when imaging findings, histological features, and clinical behavior are discordant [28,70].

^18^F-FDG PET/CT provides complementary information on tumor metabolism and may serve as an imaging surrogate of dedifferentiation, proliferative activity, and biological aggressiveness. Increased FDG uptake has been associated with atypical histology, higher proliferative indices, and less favorable outcomes [43]. Although FDG PET/CT is not recommended routinely for all lung NETs, it may be considered in selected patients with atypical carcinoids, aggressive features, low or absent SSTR expression, or discordant tumor behavior [1,28,66]. Its prognostic role, however, remains insufficiently validated prospectively, and its use should therefore be individualized [28].

The rationale for combining SSTR and ^18^F-FDG PET/CT lies in their complementary biological information: SSTR imaging reflects neuroendocrine differentiation and receptor expression, whereas FDG identifies increased metabolic activity and potentially more aggressive tumor components. Recent evidence summarized by Prosperi et al. indicates that dual-tracer PET may improve biological characterization by identifying distinct molecular imaging phenotypes, including highly differentiated SSTR-positive/FDG-negative tumors and discordant patterns suggestive of increased heterogeneity [42]. This approach may support preoperative risk stratification, prognostic assessment, and treatment planning, including identification of candidates for receptor-targeted therapies. Nevertheless, available studies remain predominantly retrospective and heterogeneous, and the prognostic impact of dual-tracer PET requires prospective validation [42]. Importantly, dual-tracer PET should complement rather than replace SSTR imaging, which remains the cornerstone of functional evaluation [42].

#### 3.3.3. Imaging for Treatment Selection and Follow-Up

Current ENETS, ESMO, AIOM, and Commonwealth Neuroendocrine Tumor Research Collaboration (CommNETS) and North American Neuroendocrine Tumor Society (NANETS) recommendations support multidisciplinary and risk-adapted management, although a standardized algorithm defining when dual-tracer PET should be systematically implemented is lacking [1,28,66,71]. This unmet need supports the development of precision imaging frameworks integrating molecular imaging with quantitative approaches such as radiomics. Radiomics and artificial intelligence-based methods may extract imaging biomarkers beyond visual interpretation, potentially improving assessment of tumor heterogeneity and individualized risk, although their application in lung NETs remains investigational [12,42].

The integration of dual-tracer PET, radiomics, and clinicopathological variables may ultimately enable a more comprehensive and biologically driven characterization of pulmonary NETs, paving the way toward personalized imaging-guided management [70]. The proposed precision imaging framework integrating conventional imaging, dual-tracer PET/CT, radiomics, and clinicopathological information is summarized in Figure 1.

#### 3.3.4. Inter-Lesional Heterogeneity and Precision Imaging

Inter-lesional heterogeneity represents an important consideration in the imaging assessment of lung NETs. Different lesions within the same patient may demonstrate variable SSTR expression and glucose metabolism, potentially reflecting biological differences in differentiation, proliferation, and disease evolution. A pattern of SSTR-positive/FDG-negative lesions may coexist with lesions showing increased FDG uptake and reduced or absent SSTR expression. Such discordant patterns may have implications for prognosis, biopsy selection, treatment planning, and the identification of lesions that may not be adequately characterized by a single imaging modality.

Dual-tracer PET/CT may therefore provide complementary information beyond the assessment of the dominant or most metabolically active lesion. However, the clinical interpretation of inter-lesional heterogeneity remains dependent on the clinical context and should be integrated with histopathology, anatomical imaging, disease kinetics, and multidisciplinary evaluation. Further prospective studies are needed to determine whether imaging-defined heterogeneity can be used to guide treatment selection or predict clinical outcomes.

## 4. Key Points for Clinicians

- Contrast-enhanced CT remains the first-line imaging modality for anatomical staging and surgical planning in pulmonary neuroendocrine tumors.
- ^68^Ga-labelled somatostatin receptor PET/CT is the reference functional imaging technique for well-differentiated lung NETs, providing staging information while simultaneously evaluating receptor status and eligibility for PRRT.
- ^18^F-FDG PET/CT complements SSTR imaging by identifying metabolically aggressive disease, particularly in atypical carcinoids, rapidly progressive tumors, or lesions with reduced SSTR expression.
- Dual-tracer PET/CT may provide complementary information on tumor heterogeneity, improving biological characterization beyond conventional histopathology and supporting individualized treatment strategies.
- Discordant imaging patterns (SSTR-positive/FDG-negative versus FDG-positive/SSTR-negative) may reflect different tumor phenotypes and should be interpreted within a multidisciplinary framework.
- Radiomics is an emerging quantitative imaging approach with potential applications in tumor classification, recurrence prediction, and personalized risk stratification.
- Current evidence supports precision imaging rather than a “one-size-fits-all” imaging approach, integrating anatomical imaging, molecular imaging, and quantitative analysis according to the clinical scenario.
- Future clinical decision-making will likely rely on multimodal imaging combined with artificial intelligence and clinicopathological data to optimize individualized management.

## 5. Conclusions and Future Perspectives

Lung NETs are biologically heterogeneous neoplasms requiring a multimodal imaging approach beyond conventional anatomical assessment. Contrast-enhanced CT remains the cornerstone of anatomical staging, while molecular imaging enables non-invasive characterization of tumor biology. SSTR PET/CT provides information on receptor expression and supports selection for receptor-targeted therapies, whereas ^18^F-FDG PET/CT offers complementary information on metabolic activity, heterogeneity, and aggressiveness in selected scenarios.

Rather than competing modalities, CT, SSTR PET/CT, and ^18^F-FDG PET/CT should be considered complementary components of an integrated strategy, improving assessment of disease extent, biological behavior, prognosis, and treatment selection, and supporting the transition toward precision imaging.

Emerging approaches such as radiomics and artificial intelligence may further refine this paradigm by identifying quantitative biomarkers of tumor heterogeneity and individualized risk, although their application remains investigational. Future prospective multicenter studies should validate these biomarkers, standardize dual-tracer imaging algorithms, and assess their impact on clinically meaningful outcomes. The integration of multimodal imaging, radiomics, and artificial intelligence may increasingly support personalized management of patients with lung NETs.

## Figures and Tables

**Figure 1:**
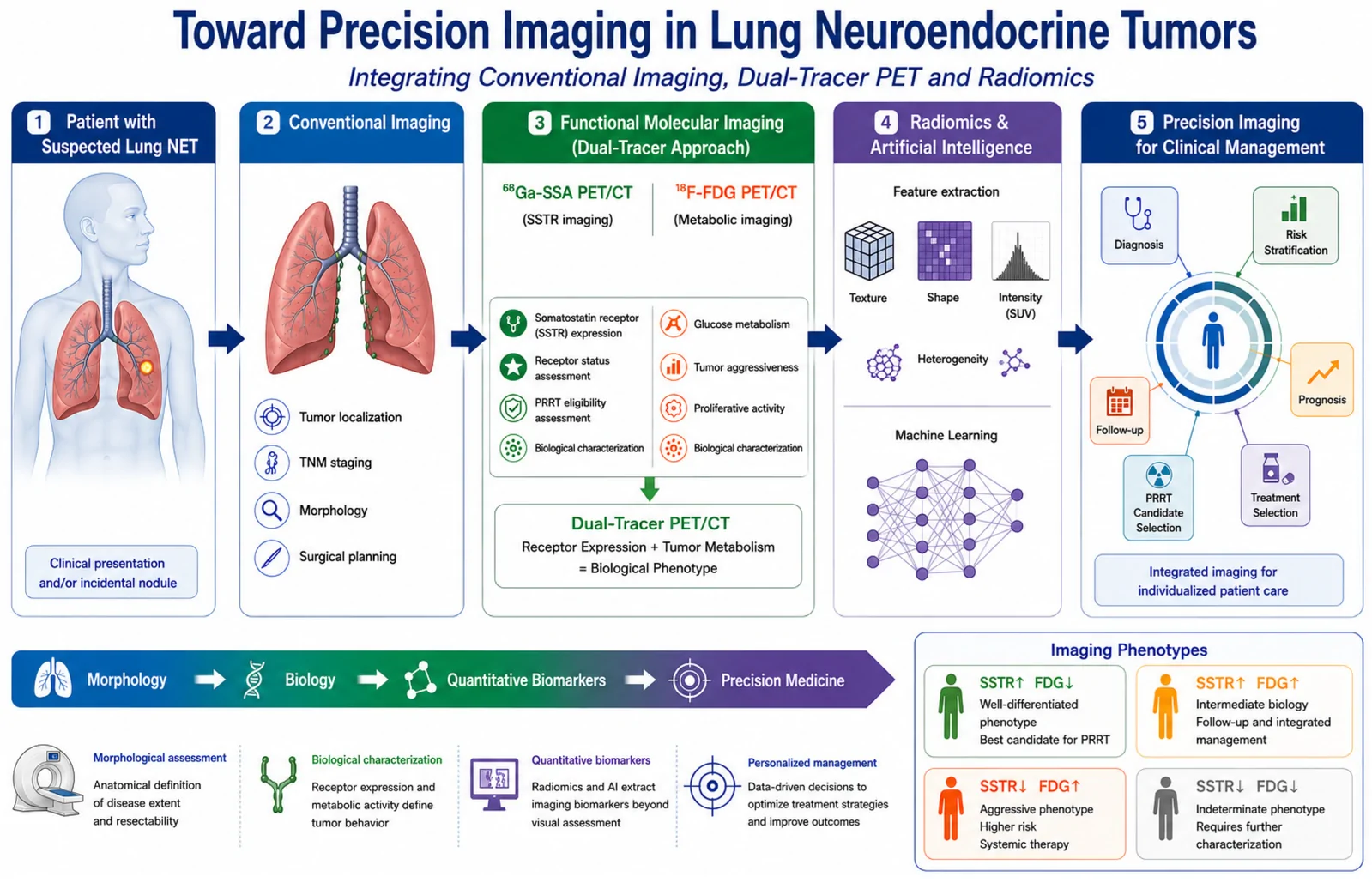
**Precision Imaging Framework for Lung Neuroendocrine Tumors.** Schematic overview of the proposed multimodal imaging framework integrating conventional contrast-enhanced CT, somatostatin receptor (SSTR) PET/CT, ^18^F- fluorodeoxyglucose (FDG) positron emission tomography/computed tomography (PET/CT), dual-tracer molecular imaging, and radiomics for lung neuroendocrine tumors (NET). The complementary information provided by anatomical, molecular, and quantitative imaging (including SUV: standardized uptake value) enables comprehensive tumor characterization, prognostic stratification, treatment selection, identification of candidates for peptide receptor radionuclide therapy (PRRT), and personalized patient management.

**Table 1:** Summary of studies evaluating functional imaging in pulmonary neuroendocrine tumors.

Imaging Modality	Study	Population	Main Findings	Potential Clinical Application
^68^Ga-SSA PET/CT	Jindal et al. [24]	Patients with lung NETs	^68^Ga-DOTA-TOC PET/CT showed a 95% detection rate and identified additional disease not detected by conventional imaging in one patient.	Tumor detection and staging.
^68^Ga-SSA PET/CT	Walker et al. [25]	Patients with indeterminate pulmonary nodules and lung cancer	Carcinoids showed higher ^68^Ga-DOTA-TATE uptake than non-neuroendocrine lung cancers. Inflammatory lesions could also demonstrate tracer uptake, limiting specificity.	Lesion characterization and assessment of SSTR expression.
^68^Ga-SSA PET/CT	Lamarca et al. [26]	Patients with lung NETs	^68^Ga-DOTA-NOC PET/CT was evaluated for diagnosis, staging, postoperative assessment, primary tumor localization, and PRRT eligibility. Imaging findings influenced clinical management in approximately one-third of patients.	Staging, treatment planning, and identification of potential PRRT candidates.
^68^Ga-SSA PET/CT	Rufini et al. [27]	Patients with lung NETs	^68^Ga-DOTA-peptide uptake correlated with immunohistochemical SSTR2 expression. Uptake was higher in typical than in atypical carcinoids.	Non-invasive assessment of SSTR expression and evaluation of eligibility for PRRT.
^18^F-FDG PET/CT	Jiang et al. [23]	Patients lung NETs	An SUVmax cutoff of 3.7 was reported for distinguishing typical from atypical carcinoids, with 73.9% sensitivity, 65.4% specificity, and an AUC of 73.3%.	Supportive characterization of tumor biology; limited diagnostic discrimination when used alone.
^18^F-FDG PET/CT	Briganti et al. [40,41]	Patients with lung NETs	The SUVmax-to-liver ratio correlated with Ki-67 in lung NETs and may help identify atypical carcinoids with higher proliferative activity. Increasing or newly developed FDG uptake during follow-up may indicate dedifferentiation or disease progression.	Assessment of tumor aggressiveness and selected follow-up scenarios.
Dual-tracer PET/CT	Jindal et al. [44]	Patients with lung NETs	The study evaluated the complementary role of ^18^F-FDG and ^68^Ga-DOTA-TOC PET/CT in differentiating typical and atypical carcinoids.	Histological characterization and complementary functional assessment.
Dual-tracer PET/CT	Venkitaraman et al. [45]	Patients with suspected bronchopulmonary NETs	^68^Ga-DOTA-TOC PET/CT demonstrated high diagnostic performance in the initial evaluation of suspected bronchopulmonary carcinoids.	Initial tumor evaluation and characterization.
Dual-tracer PET/CT	Lococo et al. [46]	Patients with lung NETs	^68^Ga-DOTA-peptide PET/CT showed higher sensitivity than ^18^F-FDG PET/CT overall, particularly in typical carcinoids, whereas FDG provided greater diagnostic accuracy in atypical carcinoids.	Complementary characterization according to histological subtype.
Dual-tracer PET/CT	Komek et al. [47]	Patients with lung NETs	The study compared ^18^F-FDG and ^68^Ga-DOTATATE PET/CT for histological subtype identification and evaluated SUVmax parameters.	Assessment of tumor phenotype and potential differentiation between typical and atypical carcinoids.
Dual-tracer PET/CT	Lococo et al. [48]	Patients with lung NETs	^68^Ga-DOTA-TOC PET/CT showed higher detection rates than FDG PET/CT overall. The diagnostic advantage of SSA imaging was more evident in typical carcinoids, whereas FDG provided greater accuracy in atypical carcinoids. Positive SSA uptake was associated with lower mitotic activity, while negative scans were more frequent in tumors with necrosis.	Histological characterization and assessment of tumor aggressiveness.
Dual-tracer PET/CT	Zidan et al. [49]	Patients lung NETs	Combined ^68^Ga-DOTA-TATE and ^18^F-FDG PET/CT identified distinct molecular imaging phenotypes based on SSTR expression and glycolytic activity.	Tumor phenotyping, risk assessment, and potential therapeutic implications, including PRRT selection.
Dual-tracer PET/CT	Deleu et al. [50]	Patients with pulmonary NETs	The value of ^68^Ga-labelled SSTR PET/CT for tumor grading and detection of disseminated disease was assessed through a pathology-based analysis and literature review.	Grading support and detection of metastatic disease.
Dual-tracer PET/CT	Albano et al. [51]	Multicentric cohort of patients with primary lung NETs	Combined ^18^F-FDG and ^68^Ga-DOTA-peptide PET/CT was evaluated for diagnostic and prognostic purposes, highlighting the complementary information provided by SSTR expression and glucose metabolism.	Integrated assessment of tumor biology, prognosis, and disease heterogeneity.

Abbreviations: FDG, fluorodeoxyglucose; NET, neuroendocrine tumor; PET/CT, positron emission tomography/computed tomography; PRRT, peptide receptor radionuclide therapy; SSTR, somatostatin receptor; SUVmax, maximum standardized uptake value.

## Data Availability

No new data were created or analyzed in this study. Data sharing is not applicable to this article.
